# Testicular artery pseudoaneurysm: a case report

**DOI:** 10.12688/f1000research.3-2.v1

**Published:** 2014-01-06

**Authors:** William P Parker, Ajay K Nangia

**Affiliations:** 1 Department of Urology, The University of Kansas Medical Center, Kansas City, KS, 66160, USA

## Abstract

This is a case of an unusual cause of a testicular mass and the clinical features associated with its presentation and management.  The patient presented with testicular pain and was found to have a testicular mass on ultrasound with a central 1cm anechoic region with arterial wave-form concerning for a pseudoaneurysm. The patient underwent orchiectomy with resolution of his symptoms. This case highlights the presentation of testicular artery pseudoaneurysm and outcome following orchiectomy.

## Case history

**Figure 1.**
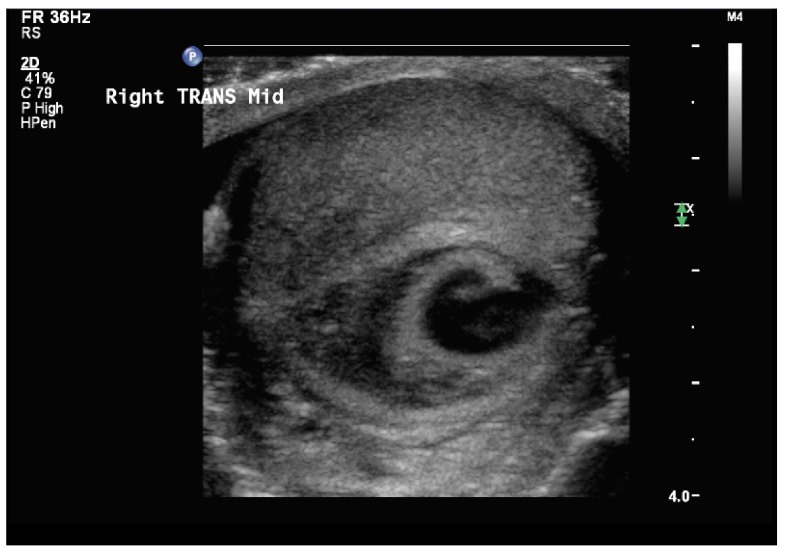
Scrotal ultrasound of the right testicle in transverse revealing a hypoechogenic lesion within the central testicle with an anechoic central core.

**Figure 2.**
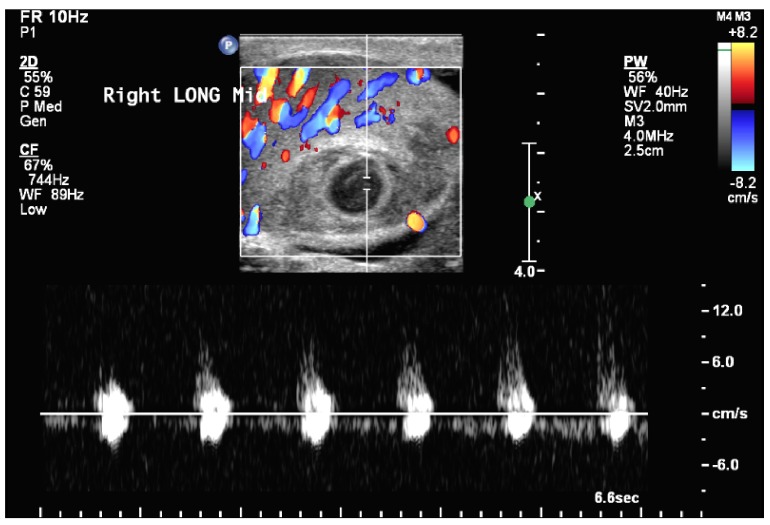
Doppler ultrasound of the right testicle in transverse reveals an arterial waveform within the center of the anechoic portion of the testicular mass.

**Figure 3.**
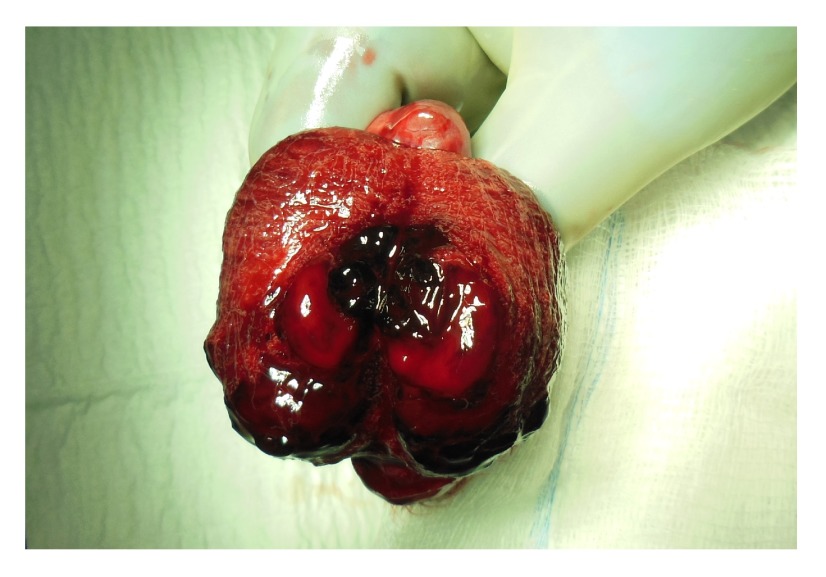
Gross section of the right testicle bi-valved with hematoma and surrounding intraparenchymal hemorrhage.

A 34 year-old Caucasian man presented to the Emergency Department at the University of Kansas Medical Center (Kansas City, Kansas) with a 1 week history of right-sided orchalgia in December of 2012. The day prior to presentation the patient was admitted at an outside institution for presumed orchitis, where he was treated with intravenous levofloxacin 500mg. During that hospitalization a scrotal ultrasound was obtained which revealed hypervascularity of the right testicle but no masses or lesions within the testicle. He was discharged the next day, but due to persistent symptoms presented to our institution. He denied any constitutional symptoms and was voiding without difficulty or irritative symptoms. He additionally denied any prior genitourinary trauma or infections including any history of orchitis, prostatitis, urinary tract infection, or sexually transmitted infection. The patient was monogamous without any high-risk behavior. The patient had no past medical history and was on no prior medications. His examination revealed a swollen and indurated right testicle without involvement of the paratesticular structures. There was no discrete testicular mass palpable. Scrotal ultrasound revealed a region of hypoechogenicity measuring 3.3cm × 2cm felt to represent an intratesticular hematoma (see Figure 1). Within this there was a 1cm central focus that demonstrated an arterial wave form with alternating reversal of flow, suggestive of a pseudoaneurysm (see Figure 2). Our differential diagnosis included abscess, testicular artery aneurysm/pseudoaneurysm, or testicular neoplasm. We counseled the patient on the ultrasonographic findings and our presumed diagnosis of testicular artery pseudoaneurysm. The patient was in a considerable amount of pain and was additionally concerned about the possibility of a testicular neoplasm and elected to undergo radical orchiectomy. Tumor markers were obtained prior to orchiectomy and were within normal limits. He tolerated the procedure without any adverse event and was discharged to home with resolution of his pain on the first post-operative morning. Pathologic evaluation confirmed the presence of significant intraparenchymal hemorrhage within a background of chronic orchitis (see Figure 3). At the time of post-operative follow-up – 2 weeks after his orchiectomy – the patient was in excellent condition with complete resolution of his pain. His surgical incision was well healed and he had no evidence of intra-scrotal pathology.

## Discussion

Intratesticular hemorrhage is frequently associated with trauma. In the absence of trauma, testicular artery aneurysm and pseudoaneurysm have been described as an infrequent source of hematoma formation. To our knowledge there have been two cases of testicular artery aneurysm and two of pseudoaneurysm reported in the literature, with etiologies of trauma ^1– 3^ and infection ^4^.

As in previous cases of testicular artery aneurysm and pseudoaneurysm, the diagnosis was established on ultrasonography. Furthermore this case describes a second scenario in which sonographically diagnosed orchitis has progressed to this clinicopathologic entity ^4^. Our management included radical orchiectomy - to rule out possible malignancy - and the patient recovered with complete resolution of his pain. This case supports orchitis as a risk factor for pseudoaneurysm formation, the use of ultrasound for the diagnosis, and the use of orchiectomy as a potential treatment in patients with unremitting pain.

Certainly the management of this case is limited by the radical treatment offered – namely orchiectomy. We did consider and offer the patient a more conservative trial of observation for possible resolution. However in the absence of overwhelming evidence to rule out malignancy we felt that radical orchiectomy would not only establish our working diagnosis, but also rule out the possibility of testicular neoplasia. Regardless, at the time of final follow-up the patient was satisfied with his course of care and the outcome.

## Consent

The patient was unable to be reached for consent and no next-of-kin information was available to contact the patient. The write-up does not contain sufficient information to identify the patient as this is a case based mainly on radiology and pathology findings. We have made numerous attempts to contact the patient and it appears that his contact information as provided at the time of treatment is no longer valid.
