# Supplementary material for: Testicular artery pseudoaneurysm: a case report
Source: F1000Res. 2014 Jan 6;3:2. [Version 1] doi: 10.12688/f1000research.3-2.v1 (PMC4118757; doi:10.12688/f1000research.3-2.v1)
Supplement: Peer review history

# Peer review: Referee response for version 1

**DOI:** 10.5256/f1000research.3344.r5013

**Published:** 2014-06-16

**Authors:** Jay Sandlow

**Affiliations:** 1 Department of Urology, Medical College of Wisconsin, Milwaukee, WI , USA

The authors describe a fairly rare case of testicular artery pseudoaneurysm, presumably secondary to orchitis. The case history is complete and the figures are well-presented. Furthermore, based on the patient’s history, age, and ultrasound findings, their management was not unreasonable. However, I would like them to describe in more detail the thought process that led to a radical orchiectomy, particularly since pseudoaneurysm was in their differential. Because the patient was of reproductive age, I wonder if it would have been possible to preserve some functioning testicular tissue. Based on the gross appearance of the specimen, it appears that at least a third of the testis was unaffected. How accurate is scrotal ultrasound in identifying a testicular artery pseudoaneurysm? Finally, looking back, is there something that the authors could have done to prevent a radical orchiectomy? This might be helpful for those of us facing this situation in the future. I think with some revisions of the Discussion, this case report should be acceptable.

I have read this submission. I believe that I have an appropriate level of expertise to confirm that it is of an acceptable scientific standard, however I have significant reservations, as outlined above.

# Peer review: Referee response for version 1

**DOI:** 10.5256/f1000research.3344.r2990

**Published:** 2014-01-24

**Authors:** Paul Turek

**Affiliations:** 1 The Turek Clinic, San Francisco, CA, USA

This case report is a classic “suspicious-looking-lesion-in-the-testicle” that is treated by removal and diagnosed definitively only after the fact, based on histology. The care provided was standard. However, besides learning about the existence of the lesion, there is no information in this case report that might help others PREVENT the extreme measure of radical orchiectomy for a benign lesion. What other diagnostic tests could have been done? Why wasn't partial orchiectomy considered in the treatment algorithm? The learning potential of this case is limited.

I have read this submission. I believe that I have an appropriate level of expertise to confirm that it is of an acceptable scientific standard, however I have significant reservations, as outlined above.

# Peer review: Referee response for version 1

**DOI:** 10.5256/f1000research.3344.r2993

**Published:** 2014-01-08

**Authors:** Nelson Bennett

**Affiliations:** 1 Institute of Urology, Lahey Clinic Medical Center, Burlington, MA, USA

This is a very nice presentation and description of a rare issue. The manuscript underscores that a complete scrotal/testicular ultrasound be carefully scrutinized. To further enhance the impact of the study, the authors may want to consider including the values of the testicular tumor markers.

Good Show.

I have read this submission. I believe that I have an appropriate level of expertise to confirm that it is of an acceptable scientific standard.
